# Cyclic ^68^Ga-Labeled Peptides for Specific Detection of Human Angiotensin-Converting Enzyme 2

**DOI:** 10.2967/jnumed.120.261768

**Published:** 2021-11

**Authors:** Matthew F.L. Parker, Joseph Blecha, Oren Rosenberg, Michael Ohliger, Robert R. Flavell, David M. Wilson

**Affiliations:** 1 Department of Radiology and Biomedical Imaging, University of California, San Francisco, San Francisco, California;; 2 Department of Medicine, University of California, San Francisco, San Francisco, California; and; 3 Department of Radiology, Zuckerberg San Francisco General Hospital, San Francisco, California

**Keywords:** COVID-19, ACE2, SARS-CoV-2, ARDS, PET

## Abstract

In this study, we developed angiotensin-converting enzyme 2 (ACE2)–specific, peptide-derived ^68^Ga-labeled radiotracers, motivated by the hypotheses that ACE2 is an important determinant of severe acute respiratory syndrome coronavirus 2 (SARS-CoV-2) susceptibility and that modulation of ACE2 in coronavirus disease 2019 (COVID-19) drives severe organ injury. **Methods:** A series of NOTA-conjugated peptides derived from the known ACE2 inhibitor DX600 were synthesized, with variable linker identity. Since DX600 bears 2 cystine residues, both linear and cyclic peptides were studied. An ACE2 inhibition assay was used to identify lead compounds, which were labeled with ^68^Ga to generate peptide radiotracers (^68^Ga-NOTA-PEP). The aminocaproate-derived radiotracer ^68^Ga-NOTA-PEP4 was subsequently studied in a humanized ACE2 (hACE2) transgenic model. **Results:** Cyclic DX-600–derived peptides had markedly lower half-maximal inhibitory concentrations than their linear counterparts. The 3 cyclic peptides with triglycine, aminocaproate, and polyethylene glycol linkers had calculated half-maximal inhibitory concentrations similar to or lower than the parent DX600 molecule. Peptides were readily labeled with ^68^Ga, and the biodistribution of ^68^Ga-NOTA-PEP4 was determined in an hACE2 transgenic murine cohort. Pharmacologic concentrations of coadministered NOTA-PEP (blocking) showed a significant reduction of ^68^Ga-NOTA-PEP4 signals in the heart, liver, lungs, and small intestine. Ex vivo hACE2 activity in these organs was confirmed as a correlate to in vivo results. **Conclusion:** NOTA-conjugated cyclic peptides derived from the known ACE2 inhibitor DX600 retain their activity when N-conjugated for ^68^Ga chelation. In vivo studies in a transgenic hACE2 murine model using the lead tracer, ^68^Ga-NOTA-PEP4, showed specific binding in the heart, liver, lungs and intestine—organs known to be affected in SARS-CoV-2 infection. These results suggest that ^68^Ga-NOTA-PEP4 could be used to detect organ-specific suppression of ACE2 in SARS-CoV-2–infected murine models and COVID-19 patients.

## Visual Abstract

**Figure:**
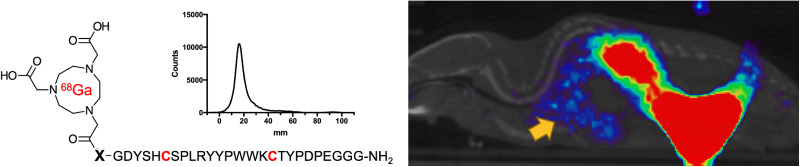

The novel severe acute respiratory syndrome coronavirus 2 (SARS-CoV-2) has had profound effects on global health, especially in the United States, the country with the largest number of confirmed coronavirus disease 2019 (COVID-19) cases and associated deaths. Many of these patients progress to acute respiratory distress syndrome (ARDS) respiratory failure with widespread injury of the lungs. The underlying mechanisms include diffuse alveolar damage, surfactant dysfunction, and immune cell activation (1–3). Many pathologic conditions can cause this convergent picture, including both bacterial and viral infections. These causes of ARDS likely share dysfunction of the renin-angiotensin system, especially loss of angiotensin-converting enzyme 2 (ACE2) function (4–8). ACE2 is a transmembrane protein that functions as an angiotensin receptor chaperone. The roles of ACE2, ACE, and angiotensin II are highlighted in Figure 1A, which describes dual functions of the renin-angiotensin system with opposing effects on cardiovascular biology (9). In this pathway, ACE2 performs an important regulatory role, converting angiotensin II to angiotensin 1–7, which causes vasodilatation and has antiinflammatory effect, unlike activation of angiotensin receptor, which will lead to vasoconstriction, higher blood pressure, and inflammation (potentially ARDS) (10–13).

**FIGURE 1.**
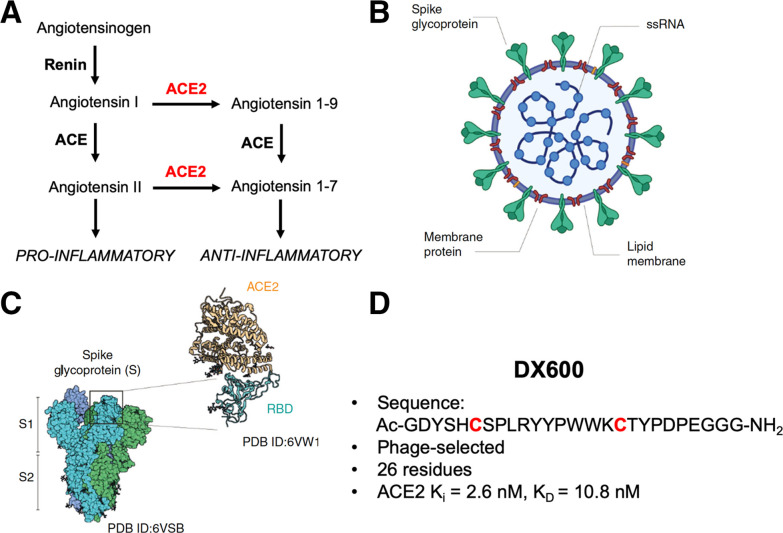
Role of ACE2 in hypertension and SARS-CoV-2 infection. (A) Renin-angiotensin system, with role of ACE2 highlighted on right; ACE2 generally counters vasoconstrictive pathway initiated by formation of angiotensin II. (B) Simplified structure of SARS-CoV-2 virus indicating spike glycoprotein that interacts with ACE2 and other host proteins. (C) Structural (cryogenic electron microscopy, x-ray crystallography) and atomic force microscopy elucidating interaction between spike protein S1 subunit and ACE2. S1 binds to ACE2 site remote to its active site, which is targeted by inhibitory peptides described in this article. (Adapted from (*18*).) (D) Characteristics of 26-residue DX600 peptide. This peptide contains 2 cysteine residues, used for cyclization via disulfide bridge formation. DX600 was discovered via phage display and shown by Huang et al. (*30*) to be a potent ACE2 inhibitor, with a calculated dissociation constant of 10.8 nM and specificity for ACE2 vs. ACE and carboxypeptidase A. K_D_ = dissociation constant; K_i_ = inhibition constant; PDB = protein data bank; RBD = receptor-binding domain; ssRNA = single-stranded ribonucleic acid.

Although several recent papers suggest that other mammalian transmembrane proteins (e.g., CD147 and CD26) allow SARS-CoV-2 to infect different cell types (14,15), ACE2 is the main point of entry of the virus into host cells (Fig. 1B). This process depends on this receptor as well as on its spike (S) protein, with cryogenic electron microscopy structures and x-ray crystal structures of the complex recently described, as well as characterization of the complex via atomic force microscopy (Fig. 1C) (16–18). This protein has 2 subunits: S1, containing receptor-binding domains, and S2, which is responsible for membrane fusion. The receptor-binding domains can mimic the ACE2 interaction with angiotensin receptor (hydrophobic and strong electrostatic interactions, including π–π, and cation–π) and gain entry via strong noncovalent attachment to ACE2 in the angiotensin receptor binding site (19). Three recent cryogenic electron microscopy structure studies demonstrated that SARS-CoV-2 spike protein binds directly to ACE2 and that the SARS-CoV-2 spike protein likely recognizes humanized ACE2 (hACE2) with even higher binding affinity than the spike from SARS-CoV (20–22). This binding was suggested to alter virus configuration and expose a cleavage site on S2, resulting in host protease cleavage (mainly by transmembrane protease/serine subfamily member 2), allowing the virus to enter the cell (23). This mechanism was recently supported by a cryogenic electron microscopy structure postfusion analysis that showed structural and conformational rearrangements of the S-protein compared with its prefusion structure (24).

To investigate SARS-CoV-2 susceptibility, and organ-specific suppression of ACE2 in COVID-19, new ACE2-specific imaging methods would be profoundly helpful. A key hypothesis in COVID-19 is that binding of SARS-CoV-2 to ACE2 results in downregulation of this beneficial enzyme, as observed for the original SARS-CoV virus from 2003, which also depends on ACE2 for viral entry. At that time, researchers in the Penninger lab found that in preclinical models of acute lung injury, the viral S-protein itself resulted in loss of normal ACE2 function, contributing to severe disease (25). After this outbreak, several ACE2-specific small molecules and peptides were discovered, motivating our design of active-site–targeted, high-affinity PET tracers. The reported ACE2-specific ligands, generally characterized by their ACE2 half-maximal inhibitory concentration (IC_50_), included the peptide DX600 discovered via phage display (26–31). The DX600 sequence is shown in Figure 1D. In this article, we report development of ACE2-specific PET radiotracers (^68^Ga-NOTA-PEP) derived from this sequence. We anticipate that ACE2-specific PET could help evaluate which systems are most targeted by SARS-CoV-2 infection, the timing of disease, and how ACE2 modulation correlates with ARDS susceptibility and other organ injury. Determining ACE2 expression noninvasively would also help us to better understand differential susceptibility to SARS-CoV-2 based on age, sex, and the use of common antihypertensive medications. Recent work has also highlighted the role of ACE2 in a large number of organs beyond the lungs, including the heart, kidneys, and gastrointestinal system (32–37). These other organ systems are affected in COVID-19 with devastating consequences. We therefore believe that the information gleaned from ^68^Ga-NOTA-PEP4 or some other in vivo ACE2 sensor will potentially be helpful in COVID-19 treatment, via either exogenous ACE2 (4,38) or some other therapy.

## MATERIALS AND METHODS

### Peptides

The DX600-derived peptides studied were obtained from AnaSpec as a custom synthesis, fully characterized by high-performance liquid chromatography (HPLC) and mass spectrometry. These peptides were radiolabeled without additional modification. Complete documentation is provided in the supplemental materials (available at http://jnm.snmjournals.org).

### ACE2 Inhibition Assay

Six DX600-derived peptides, named NOTA-PEP1 to NOTA-PEP6 (cyclic vs. noncyclic, with triglycine, aminocaproate, and polyethylene glycol linkers), were studied using a commercially available ACE2 inhibition assay according to the manufacturer’s instructions (SensoLyte 390 ACE2 Activity Assay Kit *Fluorimetric*, AS-72086; AnaSpec). Each peptide inhibitor was first tested at 4 concentrations. Initial velocities were determined relative to the inhibitor free reaction. Subsequently, IC_50_ values were derived from nonlinear fits of saturation curves of a 6-point dilution series of peptide inhibitors.

### ^68^Ga-Peptide Synthesis

Full descriptions of radiochemical syntheses, as well as the analytic techniques used, are provided in the supplemental materials. Unless otherwise noted, all reagents were obtained commercially and used without further purification. ^68^Ga-gallium chloride was generated in the University of California, San Francisco (UCSF), radiopharmaceutical facility by elution from an ITG germanium–gallium generator. To generator-eluted ^68^Ga-Cl_3_ in a 4-mL dilute HCl solution was added the indicated NOTA-PEP precursor (80 μg) in pH 5 sodium acetate buffer solution (160 μL). The mixture was heated for 15 min at 90°C. The reaction was monitored by thin-layer chromatography (TLC) performed on cellulose filter paper developed in phosphate-buffered saline. Free gallium migrates to the solvent front (∼90 mm), and bound gallium remains at the origin (∼20 mm). Crude TLC data were obtained for all ^68^Ga-NOTA-PEP peptides to determine percentage chelation; the lead peptide, ^68^Ga-NOTA-PEP4, was purified using a preconditioned C18 Sep-Pak cartridge and characterized by analytic HPLC. Stability of ^68^Ga-NOTA-PEP4 was evaluated in phosphate-buffered saline, mouse serum, and human serum in preparation for animal studies.

### Small-Animal PET/CT Imaging

All animal procedures were approved by the UCSF Institutional Animal Care and Use Committee, and all studies were performed in accordance with UCSF guidelines regarding animal housing, pain management, and euthanasia. hACE2 recombinant mice (B6.Cg-Tg(K18-ACE2)2Prlmn/J, 034860) were obtained from Jackson Laboratory, aged 6–10 wk (39–41).

For single-time-point imaging, a tail-vein catheter was placed in mice (*n* = 8) under isoflurane anesthesia. Approximately 13 MBq of ^68^Ga-NOTA-PEP4 were injected via the tail-vein catheter. The animals were placed on a heating pad to minimize shivering. They were allowed to recover and micturate, and at 75 min after injection they were placed back under isoflurane anesthesia. At 90 min after injection, the animals were transferred to an Inveon small-animal PET/CT system (Siemens) and imaged using a single static 25-min PET acquisition followed by a 10-min CT scan for attenuation correction and anatomic coregistration. No adverse events were observed during or after injection of any compound. Anesthesia was maintained during imaging using isoflurane.

For inhibition (blocking) studies (*n* = 8), the protocol was identical to that above but cold NOTA-derived inhibitory cyclic peptide (NOTA-PEP4) (10 mg/kg dose) was coadministered with ^68^Ga-NOTA-PEP4 in buffered saline.

For dynamic imaging, the protocol was similar to that above except tail-vein administration of 13 MBq of ^68^Ga-NOTA-PEP4 was performed simultaneously on a cohort of 4 animals positioned on the scanner bed for PET imaging. PET imaging data were collected beginning at the moment of injection for 90 min followed by a 10-min CT scan.

### Ex Vivo Analyses of Mice

On completion of imaging, the mice were sacrificed and biodistribution analysis performed. γ-counting of harvested tissues was performed using a Hidex automatic γ-counter. Organs were also harvested from a separate cohort of mice for an ACE2 activity assay. The tissues were homogenized, and aliquots were used for protein concentration using a standard Bradford assay. Additional tissue aliquots were used as the source of ACE2 in a commercially available ACE2 assay (AnaSpec). The initial velocities were normalized relative to muscle tissue. Relative activities are reported as the relative initial velocity per gram of protein.

### Data Analysis and Statistical Considerations

For syntheses, radiochemical yields incorporate decay correction for ^68^Ga (half-life, 68 min). All in vivo PET data were viewed using open-source Amide software (www.amide.sourceforge.net). Reported static (single-time-point) data reflect γ-counting of harvested tissues. For dynamic data, uptake was quantified by drawing spheric regions of interest (5–8 mm^3^) over indicated organs on the CT portion of the exam and expressed as percentage injected dose per gram. All statistical analysis was performed using Microsoft Excel. Data were analyzed using an unpaired 2-tailed Student *t* test. All graphs are depicted with error bars corresponding to the SEM.

## RESULTS

### NOTA-Conjugated, Cyclic Peptides Targeting the ACE2 Active Site Retain Their Potency Relative to the DX600 Parent Compound

**FIGURE 2.**
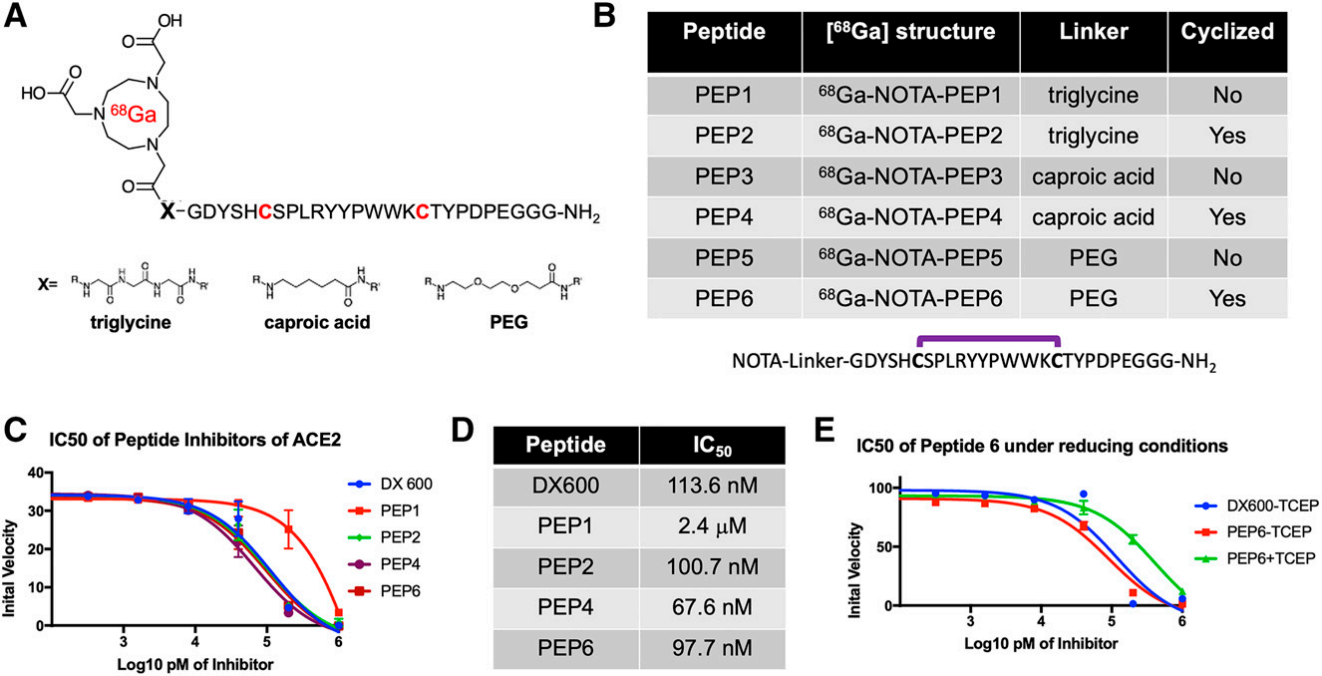
Discovery of DX600-derived, NOTA-conjugated cyclic peptide inhibitors of ACE2 from small library. (A) General ^68^Ga-peptide structure pursued. Peptides studied had N-terminal NOTA chelating group, triglycine/caproic acid/polyethylene glycol (PEG) linkers with varying degrees of hydrophobicity and hydrogen-bonding, and +/− cyclization via cysteine residues highlighted in red. (B) Identity of 6 NOTA-conjugated peptides studied. (C) Cyclic peptides demonstrated greater potency than their linear counterparts, as highlighted by initial ACE2 velocities seen with increasing inhibitor concentrations. All cyclic peptides (NOTA-PEP2, NOTA-PEP4, NOTA-PEP6) had similar profiles to parent peptide DX600, in contrast to linear peptide NOTA-PEP1. (D) ACE2 IC_50_ values calculated from these data. Of note these IC_50_ values are significantly higher than inhibition constants reported by Huang et al. for DX600, likely reflecting differences in assays used. However, NOTA-conjugated cyclic derivatives had no loss of potency relative to DX600 parent. (E) Effects of cyclization highlighted in separate ACE2 assay using tris(2-carboxyethyl)phosphine (TCEP) to reduce disulfide bridges in NOTA-PEP6.

On the basis of our hypothesis that potent peptide-derived ACE2 inhibitors, modified with linkers/chelating groups, will retain their activity and specificity, several NOTA-modified peptide-derived ACE2 inhibitors derived from the DX600 sequence (30) (inhibition constant, 2.8 nM; dissociation constant, 10.8 nM) were synthesized and screened for ACE2 inhibition. These were synthesized via Fmoc-protected linkers and N-capping NOTA reagents (Figs. 2A and 2B). The general structure pursued was a NOTA-linker peptide with 3 different linkers used, conferring varying degrees of hydrophobicity and hydrogen bonding: triglycine, polyethylene glycol, or caproic acid. These were synthesized using standard Fmoc solid-phase synthesis (AnaSpec) (42) with purity and identity confirmed by HPLC and mass spectrometry. Because DX600 contains 2 cysteine residues, a cyclized set of peptides was also synthesized via disulfide bridge formation (43). When these compounds were compared with the parent DX600 peptide in a commercially available fluorometric ACE2 inhibition assay (AnaSpec), all 3 cyclic peptides (NOTA-PEP2, NOTA-PEP4, and NOTA-PEP6) showed ACE2 inhibition nearly identical to DX600 (Figs. 2C and 2D). In other words, the N-terminal modification caused no loss of inhibitory activity when compared with the parent peptide, and in fact the cyclic peptide NOTA-PEP4 was a slightly better ACE2 inhibitor than DX600. In contrast, the linear derivatives showed much lower activity, which may result from a solution confirmation for which the NOTA interferes with ACE2 active site binding. To further evaluate this loss of potency, we studied ACE2 inhibition using a cyclic NOTA-PEP6 with and without addition of the reducing agent tris(2-carboxyethyl)phosphine, which was confirmed to reduce the disulfide bridge in the cyclic peptide (producing the linear NOTA-PEP5) (Fig. 2E; Supplemental Figs. 1 and 2). As anticipated, addition of tris(2-carboxyethyl)phosphine markedly increased the observed ACE2 IC_50_.

### Radiosyntheses of ^68^Ga-NOTA-PEP Peptides Are Efficient

**FIGURE 3.**
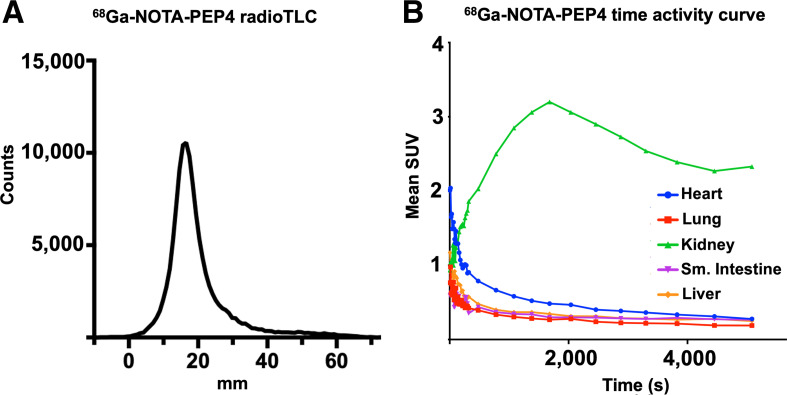
Radiosynthesis and in vivo dynamic characterization of ^68^Ga-NOTA-PEP4. On the basis of IC_50_ data, NOTA-PEP4 was chosen for subsequent radiolabeling with ^68^Ga. (A) Our optimized radiosynthesis yielded desired ^68^Ga-NOTA-PEP4 in greater than 95% radiochemical purity. (B) Dynamic small-animal PET/CT in hACE2 transgenic mice was used to generate organ-specific time–activity curve, identifying later time points as generating stable ^68^Ga signals. TLC = thin-layer chromatography.

Promising ACE2 inhibition results for NOTA-conjugated cyclic peptides were followed with radiolabeling of peptides with ^68^Ga (Supplemental Fig. 3) (44). Crude radiochemical yields of the desired ^68^Ga-peptide chelate were more than 80% in all cases by TLC. Most synthetic efforts focused on optimizing the radiosynthesis of the lead inhibitor, ^68^Ga-NOTA-PEP4. ^68^Ga-NOTA-PEP4 was synthesized in 30 min from generator-eluted ^68^Ga-Cl_3_ in a 4-mL dilute HCl solution. The precursor (80 μg) was added as a pH 5 acetate buffer solution (160 μL) and heated for 15 min at 90°C. The crude mixture was purified via a preconditioned C18 Sep-Pak cartridge, resulting in ^68^Ga-NOTA-PEP4 with more than 99% radiochemical purity as determined by radio-TLC (Fig. 3A) and HPLC (Supplemental Fig. 4). The decay-adjusted radiochemical yield of ^68^Ga-NOTA-PEP4 was 63.2% ± 6.4% (*n* = 8), with an approximate molar activity greater than or equal to 15.6 GBq/μmol. In preparation for animal studies, stability of ^68^Ga-NOTA-PEP4 was confirmed in phosphate-buffered saline, mouse serum, and human serum (Supplemental Fig. 5).

**FIGURE 4.**
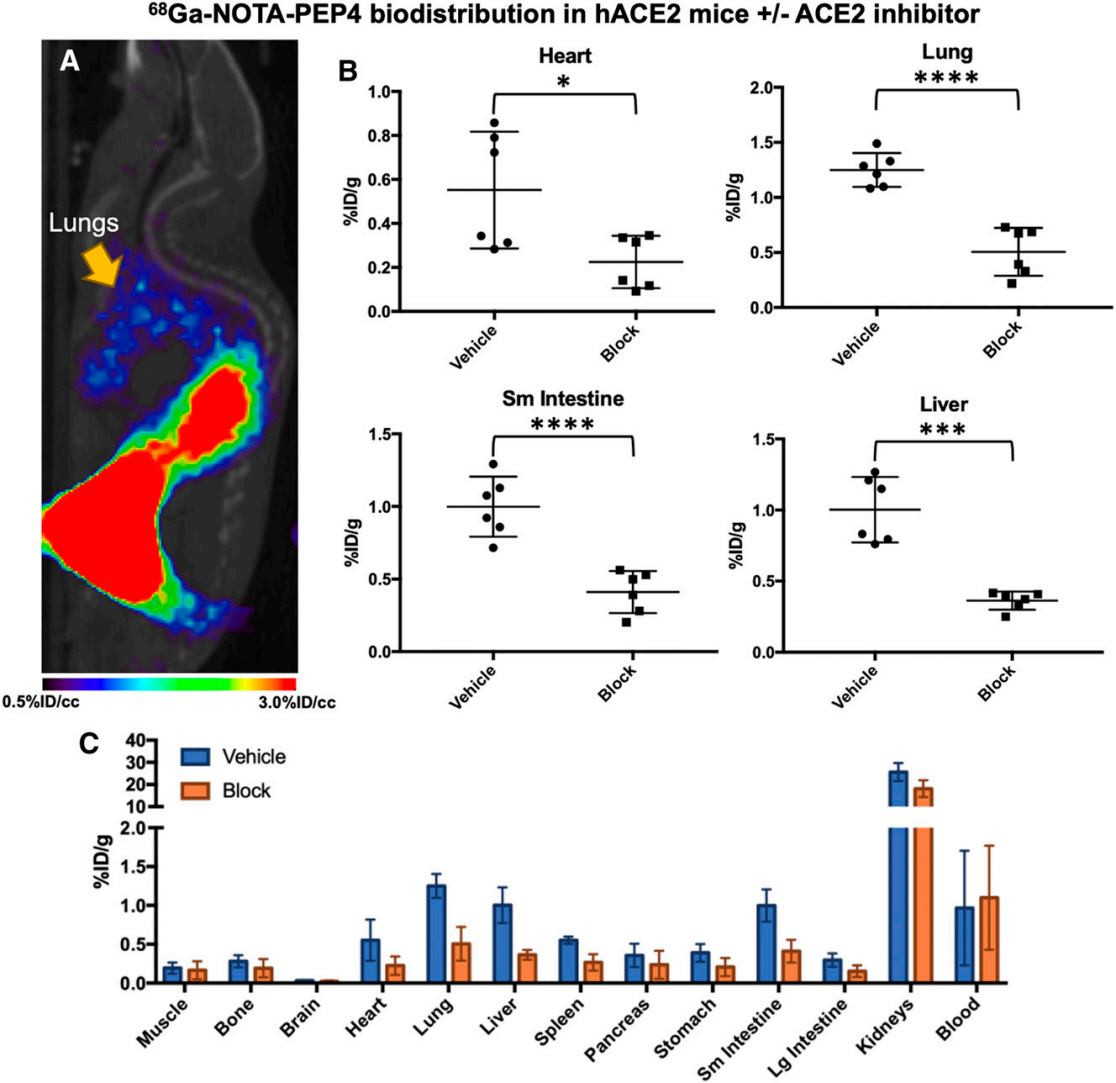
In vivo biodistribution studies of ^68^Ga-NOTA-PEP4 in hACE2 transgenic mice, demonstrating modulation of signals with pharmacologic dose of ACE2 inhibitor. (A) Small-animal PET/CT image from static acquisition highlighting signal corresponding to lungs, which is of exceptional interest in SARS-CoV-2 infection. (B) Biodistribution of ^68^Ga-NOTA-PEP4 in heart, lungs, liver, and small intestine, with and without presence of ACE2 inhibitor. Significant blocking (unpaired Student *t* test) of ^68^Ga-NOTA-PEP4 was seen in heart, lungs, liver, and small intestine, organs implicated in COVID-19. (C) Full biodistribution studies. Highest signals were observed in kidneys, but observed percentage injected dose (%ID)/g was not significantly lower in presence of ACE2 inhibitor. Therefore, renal signals are felt to represent primary route of excretion. *P ≤ 0.05. **P ≤ 0.01. ***P ≤ 0.001. ****P ≤ 0.0001.

### ^68^Ga-NOTA-PEP4 Signals in the Lungs, Heart, Small Intestine, and Liver of hACE2 Transgenic Mice Are Attenuated with Coadministration of Inhibitory Cyclic Peptide

Having developed a radiosynthesis of ^68^Ga-NOTA-PEP4, we sought to further validate the tracer in a transgenic, hACE2 murine model. The *K18-hACE2* transgenic mice express hACE2 under the control of the human keratin 18 promoter, which directs expression to epithelia, including airway epithelial cells where infections typically begin (39). Preliminary studies available from The Jackson Laboratory website, and recently published studies (45), have shown that *K18-hACE2* transgenic mice develop dose-dependent disease phenotypes when infected intranasally with SARS-CoV-2, with high doses resulting in ARDS/death analogous to that observed in some COVID-19 patients. Male Tg(K18-ACE2)2Prlmn/J hemizygous mice (*n* = 4; The Jackson Laboratory) were initially injected with 13.0 MBq of ^68^Ga-NOTA-PEP4, and dynamic imaging was performed to identify optimum single-time-point imaging. Region-of-interest analysis of dynamic data was focused on organs known to be affected in SARS-CoV-2 (Fig. 3B; Supplemental Fig. 6). Region-of-interest analysis of the images demonstrated prompt clearance from the blood pool with accumulation in the kidneys, as expected for a small-peptide tracer.

Next, we performed an imaging and biodistribution study to show that ^68^Ga-NOTA-PEP4 demonstrates specific uptake in tissues with increased expression of ACE2 (Fig. 4). To demonstrate specificity of uptake, blocking with excess cyclic NOTA-PEP inhibitory peptide was used. With blocking, significant reductions in cyclic ^68^Ga-NOTA-PEP4 were seen in the heart (2.5-fold, *P* = 0.0203), lung (2.5-fold, *P* < 0.0001), liver (2.8-fold, *P* < 0.0001), and small intestine (2.4-fold, *P* = 0.0002). ACE2 activity in these organs was subsequently confirmed via harvested organs in a separate hACE2 cohort (*n* = 3, Supplemental Fig. 7). Taken together, these data demonstrate that ^68^Ga-NOTA-PEP4 can specifically bind to tissues with high ACE2 expression.

## DISCUSSION

The novel COVID-19 has spread rapidly throughout the world, with the highest number of confirmed cases and deaths in the United States. Both biochemical studies and published cryogenic electron microscopy structures have shown that the spike protein (S-protein) of SARS-CoV-2 predominantly uses hACE2 for viral entry, resulting in suppression of this enzyme as seen in SARS-CoV (25,46). Additional recent publications have highlighted the possibility that the lower ACE2 activity seen with SARS-CoV-2 infection may be responsible for the physiologic effects incurred, analogous to what was seen with the original SARS-CoV (25). These observations support recombinant ACE2-derived therapies as a way to treat COVID-19, via 2 mechanisms: by replenishing protective ACE2 function and by serving as a decoy receptor for the virus. These therapeutic effects, the differential susceptibility of individuals (based on age, comorbidities) to COVID-19, and the organ-specific effects of SARS-CoV-2 are all potentially addressed by an ACE2-specific imaging method. We therefore sought a PET tracer derived from known inhibitor structures, via modification of the known ACE2 inhibitory peptide DX600 with ^68^Ga.

Because inhibitor-derived structures modified for PET do not necessarily recapitulate the potency of their parent compounds, our first efforts were focused on the cold NOTA-conjugated DX600-derived peptides, derived from triglycine, caproic acid, and polyethylene glycol linkers. Gratifyingly, the DX600-derived cyclic peptides that were studied all showed ACE2 activity similar to the parent peptide. In contrast, the linear versions were relatively inactive, which may reflect conformational effects. The calculated IC_50_ of DX600 (standard included in AnaSpec assay kit) was more than 1 order of magnitude higher than the inhibition constant reported by Huang et al. (30), likely reflecting numerous experimental differences (e.g., enzyme concentration and activity). We therefore considered the IC_50_ of the NOTA-derived peptides relative to that of DX600 to be the most important determinant of successful PET probe development. Indeed, our lead cyclic peptide, NOTA-PEP4, had an IC_50_ lower than that of the DX600 parent, motivating the radiolabeling of NOTA-PEP4 for subsequent imaging studies.

A high-yield and efficient synthesis of ^68^Ga-NOTA-PEP4 was developed with the tracer applied to an hACE2 transgenic model. Our studies coinjecting a pharmacologic concentration of NOTA-PEP inhibitor with ^68^Ga-NOTA-PEP4 showed significant attenuation of PET signals in the lungs, liver, heart, and small intestine, suggesting that these signals were related to ACE2 expression. Consistent with this observation, ex vivo tissue-specific ACE2 activity was observed in these organs, which are affected in COVID-19 (47,48). Modulation of ^68^Ga-NOTA-PEP4 using an ACE2 inhibitor also suggests that changes in ACE2 expression can be detected noninvasively. Additionally, ex vivo tissue analysis showed metabolically active ACE2 expression in the kidneys despite the absence of strong blocking. The tissue accumulation of ^68^Ga-NOTA-PEP4 in the kidneys suggests a dominant renal excretion pathway, complicating our ability to detect hACE2 in this tissue (49). In other words, high background signal due to the normal excretion pathway of ^68^Ga-NOTA-PEP4 may represent a limitation of this method to detect ACE2 activity in the kidney. In the future, hACE2 expression–specific ^68^Ga-NOTA-PEP4 signals versus background excretion need to be further clarified, perhaps using ACE2 knockout animals (50) in addition to the inhibitory studies described in this article.

The in vivo studies performed also reflect a limitation of most academic centers in the United States; specifically, few facilities have a small-animal PET/CT imaging system compatible with biosafety level 3. Future molecular imaging of live SARS-CoV-2 (a biosafety level 3 organism) and its host effects will therefore require collaborative work with those few centers able to accommodate these studies (51). Given the history of ACE2 with respect to SARS-CoV (the 2003 SARS coronavirus) and ARDS, we expect that new ACE2-specific PET tools will be relevant beyond the current pandemic. We are partially motivated by data indicating that zoonotic infections, especially coronavirus-related, are on the rise (52). The incidence of emerging and reemerging zoonotic disease is increasing in many parts of the world, with animal viruses able to cross species barriers to infect humans; it appears likely that ACE2 will be relevant in future pandemics. Better understanding ACE2 suppression, and differential susceptibility to SARS-COV-2, will help us better treat COVID-19 and other diseases for which ACE2 plays a critical role.

## CONCLUSION

Our study shows that the ACE2 active site–targeted inhibitor DX600 can be modified for PET via NOTA/linker modification, without loss of activity for cyclized peptides. All peptides studied are readily radiolabeled with ^68^Ga. In an hACE2 transgenic murine model, the lead radiotracer, ^68^Ga-NOTA-PEP4, shows dominant excretion from the kidneys, with attenuated uptake in the lungs, liver, heart, and small intestine when an ACE2 inhibitor is coadministered. These results suggest that modulation of ACE2, as occurring in SARS-CoV-2 infection, can be detected using ^68^Ga-NOTA-PEP4 or related approaches. Future studies include application of ^68^Ga-NOTA-PEP4 to SARS-CoV-2–infected hACE2 mice.

## DISCLOSURE

This work was supported by NIH R01 EB024014 and R01 EB025985 and by the UCSF Resource Allocation Program. No other potential conflict of interest relevant to this article was reported.

**KEY POINTS**

**QUESTION:** Can ACE2, the main receptor for SARS-CoV-2, be detected using PET?
**PERTINENT FINDINGS:** NOTA-conjugated cyclic peptides derived from the known ACE2 inhibitor DX600 retain their activity when N-conjugated for ^68^Ga chelation. In vivo studies in a transgenic hACE2 murine model using the lead tracer, ^68^Ga-NOTA-PEP4, showed specific binding in the heart, liver, lungs, and intestine—organs known to be affected in SARS-CoV-2 infection.
**IMPLICATIONS FOR PATIENT CARE:** The spatiotemporal distribution of ACE2 suppression in COVID-19 will be helpful both in understanding the disease and in developing future treatments. Specifically, the loss of normal ACE2 activity is implicated in organ dysfunction (particularly lung dysfunction), a deficit that may be addressed by recombinant ACE2 administration or some other therapy.
